# Retinopathy among young adults with Diabetes Mellitus from a tertiary care setting in Sri Lanka

**DOI:** 10.1186/1472-6823-14-20

**Published:** 2014-03-04

**Authors:** Prasad Katulanda, Yasindu C Waniganayake, Priyanga Ranasinghe, WM Udai Akalanka Wijetunga, Mahesh Jayaweera, Nishantha P Wijesinghe, Rezvi Sheriff, David R Matthews

**Affiliations:** 1 Department of Clinical Medicine, Faculty of Medicine, University of Colombo, Kynsey Road, Colombo, Sri Lanka; 2 Oxford Centre for Diabetes, Endocrinology and Metabolism, University of Oxford, Oxford, United Kingdom; 3 Ministry of Health Care and Nutrition, Colombo, Sri Lanka; 4 Department of Pharmacology, Faculty of Medicine, University of Colombo, Colombo, Sri Lanka

**Keywords:** Diabetic retinopathy, Diabetes mellitus, Rate, Young-onset diabetes, Sri Lanka

## Abstract

**Background:**

Diabetic retinopathy (DR) is one of the leading causes for complete loss of vision among working-aged adults around the world. The present study aims to evaluate the rate of DR and its risk factors among the adults with young-onset diabetes from a tertiary care setting in Sri Lanka.

**Methods:**

A consecutive sample of 1,007 individuals referred from multiple centers, were invited for the study. Ophthalmological evaluation was done, with dilated indirect ophthalmoscopy by an Ophthalmologist. Retinopathy was classified according to the International Clinical DR Disease Severity Scale. An interviewer-administered questionnaire was used to collect socio-demographic and anthropometric details. Seated blood pressure, Fasting Blood Glucose (FBG), HbA1c and urine microalbumin were also measured. Data were analysed using SPSSv14. A binary logistic regression analysis was performed in all patients, with ‘presence of DR’ as the dichotomous dependent variable and other independent covariates.

**Results:**

Sample size was 684 (response rate–67.9%), mean age was 37.1 ± 5.9 years and 36.0% were males. Mean duration of diabetes was 5.2 ± 4.0 years. Previous retinal screening had been done in 51.0% by a non-specialist doctor and in 41.5% by a consultant ophthalmologist. Rate of any degree of DR in the study population was 18.1% (Males 16.4%, Females 20.0%; P = NS). In patients with DR, majority had mild Non-Proliferative DR (NPDR) (57.2%), while 32.2% had moderate NPDR, 0.8% had severe NPDR and 9.7% had maculopathy. Mean age, duration of diabetes, systolic (SBP) and diastolic blood pressure (DBP), FBG, HbA1c and urine microalbumin levels were significantly higher amongst the patients with DR. The results of the binary logistic regression indicate that the duration of diabetes (OR:1.24), HbA1c (OR:1.19), age (OR:1.11), urine Microalbumin (OR:1.11) and DBP (OR:1.04) all were significantly associated with DR.

**Conclusions:**

In this large multi center study, nearly one in five adults with young-onset diabetes was found to have retinopathy. Age, duration of diabetes, HbA1C and urine Microalbumin levels were significantly associated with the presence of retinopathy, while HbA1c was also a significant factor determining severity. Nearly 50% of the study population has never undergone retinal screening by an ophthalmologist, highlighting the need for well organized screening programs.

## Background

Diabetic retinopathy (DR) is the most common microvascular complication of diabetes, resulting from retinal vascular damage due to poor glycaemic control [1,2]. It is one of the leading causes for complete loss of vision among working-aged adults around the world [3]. DR is the cause of blindness in approximately 2.5 million of the estimated 50 million blind people in the world [4]. Loss of productivity and reduced quality of life for the patient with DR leads to additional socio-economic burdens on the community. In patients with type 2 diabetes 50-70% have been observed to have DR after 10 years, and 90% after 30 years [5,6]. The prevalence of DR is significantly higher in type 1 than in type 2 diabetics and it is also more aggressive and accelerated in patients with type 1 diabetes [7]. Retinopathy in diabetes is known to be associated with the duration of diabetes, sub-optimal glycaemic control, presence of hypertension and diabetic nephropathy [8-10].

South Asia, commonly known as the Indian sub-continent, is home to almost one-quarter of the world’s population. Currently South Asia is experiencing a significant epidemic of diabetes with a rapid increase in prevalence over the last two decades [11]. There is conflicting evidence regarding the epidemiology of DR in South Asians. The U.K. Prospective Diabetes Study (UKPDS) showed a similar prevalence of retinopathy in South Asian, Afro-Caribbean, and white Europeans with diabetes [12]. However, several subsequent studies in UK reported a higher prevalence of retinopathy in South Asians residing in UK than in the native European population [13,14]. Genetic differences between racial groups may contribute to this differential occurrence of DR in the different ethnic groups.

The prevalence of diabetes mellitus has reached epidemic proportions in Sri Lanka. In 2005, the prevalence was nearly 11%, while 1/5th of the adult population were suffering from dysglysaemia (diabetes and pre-diabetes), furthermore it is estimated that over 1/3rd of the population may be undiagnosed [15,16]. In 1993, Fernando et al. studied 1,003 Sri Lankan patients with type 2 diabetes attending a clinic and demonstrated that 31.3% had DR and 23% had cataract [17]. Weerasuriya et al., conducted a study among newly diagnosed patients with type 2 diabetes residing in the Colombo district of Sri Lanka in 1998 and showed a 15% prevalence of DR [18]. However, there are no recent large scale multi-center studies evaluating the prevalence of DR and its associations in Sri Lankan adults with young-onset diabetes. The present study aims to evaluate the rate of DR and its risk factors among the young adult patients with diabetes from Sri Lanka.

## Methods

### Study population and sampling

In this study, a consecutive sample of 1,007 individuals was referred from; 1) the three largest government tertiary care hospitals in Colombo (National Hospital of Sri Lanka, Colombo South Teaching Hospital and Colombo North Teaching Hospital), 2) diabetes and medical outpatient clinics of few other selected government hospitals, 3) referrals from physicians or general practitioners and 4) the National Diabetes Centre in Sri Lanka. At these centers the total number of diabetic patient visits are more than 10,000/month. All young-adults, who had been diagnosed with diabetes between 16–40 years of age and who were ≤45 years at the time of the study were referred. Patients with history of glaucoma, dense cataract and corneal opacities were excluded, as it was difficult to perform retinal screening. In addition pregnant women were also not included in the study. Those providing informed written consent was recruited for the study. Recruitment was carried out during a period of 9 months, from June 2005 to February 2006. The study was conducted in accordance with the principles of the Declaration of Helsinki. Ethics approval for the study was obtained from the Ethics Review Committee, Faculty of Medicine, University of Colombo.

### Ophthalmological examination

Ophthalmological evaluation was done at the National Eye Hospital, Colombo, Sri Lanka, which is the largest tertiary care eye hospital in the country. All patients had their pupils dilated with 1% tropicamide and was examined for DR by an Ophthalmologist by indirect ophthalmoscopy using slit lamp biomicroscopy. Features identified in slit lamp examination was recorded and retinopathy was classified in to the following categories, according to the International Clinical DR Disease Severity Scale; normal, mild Non Proliferative DR (NPDR), moderate NPDR, severe NPDR, proliferative DR and clinically significant macular edema (maculopathy) (Additional file 1) [19]. The presence of retinopathy in one eye was considered a diagnosis of DR and when asymmetrical DR was present the stage of retinopathy was based on the affected eye with the more severe grade of DR.

### Data collection and definitions

Data collection was carried out at the Diabetic Research Unit, Department of Clinical Medicine, Faculty of Medicine, University of Colombo. An interviewer administered questionnaire was used to collect socio-demographic and anthropometric details including age, gender, duration of diabetes, height, weight, waist circumference and hip circumference. Height was measured using Harpenden stadiometers (Chasmors Ltd, London, UK) to the nearest 0.1 cm, according to standard methods. Body weight was measured using a SALTER 920 digital weighing scale (SALTER Ltd, Tonbridge, UK) to the nearest 0.1 kg. Body Mass Index (BMI) was calculated as weight in kilograms divided by height squared in meters (kg/m^2^). Waist circumference (WC) was measured midway between the iliac crest and the lower rib margin at the end of normal expiration and hip circumference was measured at the widest level over the greater trochanters using a plastic flexible tape to the nearest 0.1 cm. Waist to Hip Ratio (WHR) and Waist to Height Ratio (WHtR) were calculated as waist circumference divided by hip circumference and height respectively.

Seated blood pressure was measured after at least a 10-min rest with Omron IA2 digital blood pressure monitors (Omron Healthcare, Singapore). The following biochemical tests were also done; Fasting Blood Glucose (FBG) (glucose oxidase method – Hitachi 704 chemical auto-analyzer), Glycosylated haemoglobin (HPLC technique) and urine microalbumin. Hypertension was defined as systolic blood pressure > 130 mmHg and/or diastolic blood pressure > 80 mmHg and/or being on anti-hypertensive treatment. Central obesity was classified as WC > 90 cm for males and > 80 cm for females (Asian cut-offs) [20]. Obesity was defined as a BMI ≥ 27.5 kg/m^2^, based on WHO criteria for Asians [21].

### Statistical analysis

Data were analysed using SPSS v14 (SPSS Inc., Chicago, IL, USA) statistical software package. The significance of the differences between proportions and means was tested using z-test and Student’s *t*-test or ANOVA respectively. Subjects were divided in to two groups based on the presence or absence of DR. A binary logistic regression analysis was performed in all patients with ‘presence of DR’ as the dichotomous dependent variable (0 = DR absent; 1 = DR present) and age, duration of diabetes, BMI, WC, hip circumference, systolic blood pressure, diastolic blood pressure, FBG, HbA1c and urine microalbumin as the continuous independent variables. The explanatory independent variables that were associated with the dependent variable in univariate analysis (p < 0.25) were selected to be included in the regression analysis. The explanatory variables selected above were subsequently included in a binary logistic regression model, a backward elimination procedure was used and a p-value of 0.10 was considered as the cutoff for removal of variables. A similar binary logistic regression analysis with above dependant and independent variables was also performed separately for both males and females.

To evaluate factors associated with the severity of DR, patients with DR were regrouped in to the following two groups; 1) ‘mild’ disease (patients with mild NPDR) and 2) ‘moderate-severe’ disease (patients with moderate NPDR, severe NPDR and maculopathy). A binary logistic regression analysis was performed in all patients with DR, using ‘moderate-severe DR’ as the dichotomous dependent variable (0=’mild’ DR; 1=’moderate-severe DR) and duration of diabetes, systolic blood pressure, diastolic blood pressure, HbA1c and urine Microalbumin as the continuous independent variables. The explanatory independent variables were selected as in the above regression analysis. In all statistical analyses *p* < 0.05 was considered significant.

## Results

### Sample characteristics

A sample of 1,007 individuals were referred from the above centre’s and 684 consented to participate in the study (response rate – 67.9%), mean age (±SD) was 37.1 ± 5.9 years and 36.0% (n = 246) were males. The mean age of diabetes diagnosis (±SD) and duration of diabetes (±SD) were 31.9 ± 5.7 years and 5.2 ± 4.0 years (range 1–24 years) respectively. The prevalence of obesity (BMI ≥ 27.5 kg/m^2^) and central obesity (based on waist circumference) in the study population were 18.9% (n = 129) and 62.9% (n = 430) respectively. Hypertension was present in 23.1% (n = 158) of the study population. In the current study population the mean FBG was 166.5 ± 68.6 mg/dl, and the mean HbA1c was 8.1 ± 2.0% (65.0 mmol/mol). Out of all the patients in the study, previous retinal screening had been not been done in 34.1% (n = 233), and in the 65.9% who underwent previous examination it was done by only a consultant ophthalmologist in 12.7% (n = 87), by only a non-specialist doctor in 26.4% (n = 180) and by both in 26.8% (n = 183).

### Rate, clinical and biochemical correlates of DR

The rate of any degree of DR in the study population was 18.1% (n = 124). There was no significant gender difference observed in the rate of DR (Males 16.4%, Females 20.0%; P = NS). The rate of cataract was 5.1% (Males 3.4%, Females 6.1%; P = NS). In the patients with DR, majority had mild NPDR (57.2%, n = 71), while 32.2% (n = 40) had moderate NPDR, 0.8% (n = 1) had severe NPDR and 9.7% (n = 12) had maculopathy. There were no patients with PDR in the current study population. The mean age, duration of diabetes, systolic and diastolic blood pressure, fasting blood glucose, HbA1c and urine microalbumin levels were significantly higher amongst the patients with DR than those without (Table 1). However the mean weight, BMI, waist and hip circumferences were significantly lower in the patients with DR (Table 1). The duration of diabetes, HbA1c, systolic and diastolic blood pressures were significantly higher in patients with maculopathy than in patients with mild NPDR (Table 1).

**Table 1:** Association of age, clinical and biochemical parameters with diabetic retinopathy

	**Diabetic retinopathy**		p*	**Diabetic retinopathy grade**		
	**Absent**	**Present**		**Mild NPDR**	**Moderate NPDR**	**Maculopathy**
	**Mean (±SD)**	**Mean (±SD)**		**Mean (±SD)**	**Mean (±SD)**	**Mean (±SD)**
Age (years)	36.5 (±5.9)	39.8 (±4.6)	<0.001	39.1 (±5.1)	40.4 (±4.0)	40.8 (±3.0)
Duration of diabetes (years)	4.4 (±3.3)	8.5 (±4.9)	<0.001	7.9 (±4.7)^♯^	8.8 (±4.9)^§^	11.2 (±5.0)^♯,§^
Height (cm)	157.6 (±10.0)	156.2 (±8.0)	NS	155.9 (±8.0)	156.0 (±7.4)	158.4 (±10.4)
Weight (kg)	61.9 (±11.9)	57.1 (±9.6)	<0.001	57.2 (±10.2)	55.8 (±9.0)	60.4 (±8.7)
Body Mass Index (kg/m^2^)	24.8 (±4.2)	23.3 (±3.2)	<0.001	23.4 (±3.2)	22.8 (±3.4)	24.1 (±2.7)
Waist circumference (cm)	87.4 (±9.8)	85.0 (±8.4)	<0.05	84.8 (±8.6)	84.3 (±8.3)	88.7 (±5.9)
Hip circumference (cm)	96.0 (±15.5)	91.8 (±6.3)	<0.01	92.2 (±6.5)	90.6 (±6.1)	93.5 (±5.1)
Waist to hip ratio	0.91 (±0.06)	0.92 (±0.06)	NS	0.92 (±0.05)	0.93 (±0.06)	0.95 (±0.04)
Waist to height ratio	0.56 (±0.07)	0.54 (±0.05)	NS	0.54 (±0.05)	0.54 (±0.06)	0.56 (±0.04)
Systolic blood pressure (mmHg)	119.1 (±12.7)	124.2 (±17.1)	<0.001	121.3 (±16.3)^♯^	125.3 (±16.1)	138.1 (±19.4)^♯^
Diastolic blood pressure (mmHg)	74.8 (±8.9)	77.4 (±10.2)	<0.01	75.9 (±8.9)^♯^	77.9 (±11.7)	83.4 (±10.6)^♯^
Fasting blood glucose (mg/dl)	163.6 (±66.8)	184.2 (±76.5)	<0.01	180.6 (±73.5)	187.4 (±82.0)	201.9 (±76.2)
HbA1c (%) [mmol/mol]	7.9% (±2.0) [62.8]	9.0% (±1.8) [74.9]	<0.001	8.7 (±1.8)^♯^ [71.6]	9.4 (±1.6) [79.2]	10.1 (±1.2)^♯^ [86.9]
Urine Microalbumin (mg/l)^¥^	7.9 (3.6 – 26.5)	14.3 (4.2 – 53.2)	<0.05	9.8 (4.1 – 45.6)^♯^	23.3 (7.2 – 71.2)^♯^	11.5 (3.0 – 97.5)

*- patients with and without diabetes retinopathy; NPDR – Non Proliferative Diabetic Retinopathy; NS – Not Significant; ^¥^- Median and inter-quartile range; ^♯,§^values in a single row with the same superscript in the diabetes retinopathy grade are significantly different from each other (p < 0.01).

In all adults the rate of DR was significantly higher in patients who were having; a duration of diabetes > 5 years, systolic hypertension or diastolic hypertension or both, a HbA1c of > 6.5% (> 47.5 mmol/mol) and a urine Microalbumin of > 200 mg/l (Table 2). However the rate of DR was significantly lower in patients who were obese. A similar pattern was observed independently in females, but in males presence hypertension (systolic/diastolic/both) did not significantly influence the rate of DR (Table 2).

**Table 2:** Rate of retinopathy in association with anthropometric, clinical and biochemical parameters

	**Rate of diabetic retinopathy (%, [95% CI])**		
	**All adults**	**Males**	**Females**
Duration of diabetes			
≤ 5 years	9.4 (6.7 – 12.6)^§^	5.7 (2.6 – 10.6)^§^	11.5 (7.9 – 16.1)^§^
> 5 years	34.7 (28.8 – 41.0)^§^	37.0 (26.6 – 48.5)^§^	33.5 (26.4 – 41.3)^§^
Central obesity*			
Absent	19.7 (15.0 – 25.0)	16.8 (11.1 – 23.9)	23.8 (15.9 – 33.3)
Present	18.2 (14.7 – 22.1)	15.8 (9.1 – 24.7)	18.9 (14.8 – 23.6)
Obesity (BMI > 27.5 kg/m^2^)			
Absent	21.2 (17.8 – 24.9)^§^	17.8 (12.8 – 23.8)^§^	23.2 (18.8 – 28.2)^§^
Present	8.9 (4.5 – 15.3)^§^	6.3 (1.0 – 10.8)^§^	9.8 (4.6 – 17.8)^§^
Systolic hypertension (> 130 mmHg)			
Absent	16.3 (13.2 – 19.7)^§^	14.9 (10.1 – 21.0)	17.0 (13.2 – 21.4)^§^
Present	28.7 (21.1 – 37.3)^§^	20.4 (10.6 – 33.6)	34.7 (24.0 – 46.5)^§^
Diastolic hypertension (> 80 mmHg)			
Absent	17.4 (14.4 – 20.7)^§^	15.5 (10.8 – 21.3)	18.4 (14.6 – 22.7)^§^
Present	27.6 (21.5 – 36.2)^§^	20.0 (8.4 – 36.9)	32.7 (24.3 – 43.1)^§^
Hypertension^♯^			
Absent	16.6 (13.5 – 20.2)^§^	15.5 (10.4 – 21.8)	17.2 (13.3 – 21.7)^§^
Present	25.3 (21.7 – 30.8)^§^	17.9 (9.6 – 29.2)	30.8 (24.6 – 37.3)^§^
HbA1c			
≤ 6.5% (≤ 47.5 mmol/mol)	7.1 (3.7 – 12.0)^§^	1.7 (0.04 – 8.9)^§^	10.0 (5.1 – 17.2)^§^
> 6.5% (> 47.5 mmol/mol)	22.7 (19.0 – 26.7)^§^	21.7 (15.8 – 28.6)^§^	23.2 (18.6 – 28.3)^§^
Urine microalbumin			
< 20 mg/l	14.7 (11.1 – 18.9)^§^	8.8 (4.5 – 15.2)^§^	18.0 (13.2 – 23.7)^§^
20 – 200 mg/l	22.0 (15.6 – 29.5)	26.5 (16.5 – 38.6)	18.3 (10.6 – 28.4)
> 200 mg/l	41.2 (25.4 – 60.1)^§^	33.3 (17.5 – 50.1)^§^	50.0 (25.7 – 74.3)^§^

^§^Values under each variable in a single column with the same superscript are significantly different from each other (p < 0.01); ^♯^systolic hypertension or diastolic hypertension or both; ^*^waist circumference > 90 cm for males and > 80 cm for females.

### Results of the logistic regression analysis

The results of the binary logistic regression analysis in all adults using the dichotomous variable ‘presence of DR’ as the dependant factor and other independent variables are shown in Table 3. The overall model was statistically significant and the Cox & Snell R-Square and Nagelkerke R Square values were 0.232 and 0.384 respectively. The results indicate that the duration of diabetes (OR: 1.24), HbA1c (OR: 1.19), age (OR: 1.11), urine Microalbumin (OR: 1.11) and diastolic blood pressure (OR: 1.04) all were associated with significantly increased risk of developing DR (Table 3). Duration of diabetes, HbA1c and urine Microalbumin were also associated with developing DR in both males and females independently (Table 3). However age, systolic and diastolic blood pressure did not show a consistent relationship when both genders were considered separately. In the regression analysis evaluating factors associated with the severity of DR, only HbA1c (OR: 1.38) and systolic blood pressure (OR: 1.03) were significantly associated with the presence of ‘moderate-severe DR’.

**Table 3:** Binary logistic regression analysis on presence of retinopathy in all adults, males and females

	**Odds ratio (95% CI)**		
**Co-variants**	**All adults**	**Male**	**Female**
Age (years)	1.11 (1.04 – 1.18)^#^	NA	1.11 (1.02 – 1.21)^§^
Duration of diabetes (years)	1.24 (1.15 – 1.33)^*^	1.56 (1.32 – 1.82)^*^	1.19 (1.10 – 1.29)^*^
Systolic blood pressure (mm Hg)	NA	NA	1.02 (1.00 – 1.05)^§^
Diastolic blood pressure (mm Hg)	1.04 (1.01 – 1.07)^§^	NA	NA
HbA1c (%)	1.19 (1.04 – 1.36)^#^	1.32 (1.05 – 1.64)^§^	1.20 (1.10 – 1.30)^#^
Urine Microalbumin (mg/l)	1.11 (1.10 – 1.21)^§^	1.10 (1.05 – 1.15)^§^	1.16 (1.10 – 1.22)^§^

*- p < 0.001, #- p < 0.01, §- p <0.05; NA – Not Associated.

## Discussion

To our knowledge this is the largest study from the South Asian region among adult patients with young-onset diabetes. In this cohort of patients there was a higher proportional prevalence of type 2 diabetes (nearly 95%), while autoimmune diabetes was much less common than in the European populations [22]. Type 2 diabetes occurs at a younger age in South Asians when compared to Europeans [23]. Due to their longer exposure to hyperglycemia, patients with young‒onset type 2 diabetes are at an increased risk of developing diabetes related complications. In addition there is considerable evidence to support the fact that complications are commoner amongst South Asian patients with diabetes [24]. The decline in glycaemic control over time is also much more rapid among South Asians [25]. Hence, diabetes among South Asians represents a differential disease with a much more aggressive progression than in other ethnic groups. A significant majority of studies evaluating complication and risk factors in young-adult South Asians with diabetes are amongst the South Asian immigrant populations living in developed countries. Changes in lifestyles associated with migration could play a role in the pathogenesis of diabetes and its complications in these migrant South Asians living in western developed countries. Hence, the current study is important as we look at the rate and risk factors for developing DR a common complication of diabetes, in a large cohort of ethnic South Asian adults with young-onset diabetes residing in South Asia. In addition, from a public health perspective adults with young-onset diabetes represents a group of patients in whom early risk factor interventions may significantly attenuate the risk of complications.

In the current cohort the rate of DR was 18.7% (Males 16.4%, Females 20.0%), with a majority of patients having either mild (57.2%) or moderate (32.2%) NPDR. A similar prevalence was observed among South Asians residing in UK (17.6%), while this prevalence was significantly higher than the native Europeans (7.9%) with young-onset type 2 diabetes [13]. However, our rate was higher than the rate observed in similar studies among young adults with diabetes from India (5.3%), Malaysia (10.0%) and China (15.1%) [26]. Although nearly one in five Sri Lankan adults with young-onset diabetes was found to have DR (after a mean duration of 5 years from diagnosis), a significant majority (89.4%) are having only mild degrees of DR. This rapid onset of DR in young patient with type 2 diabetes emphasizes the importance of careful and regular ophthalmological evaluation in all patients from the time of diagnosis. Furthermore the high rate of mild DR is important since appropriate measures instituted at these early stages can either halt or slowdown the progression of disease. However, in the present study previous retinal screening has been done only in 40.7% by a specialist ophthalmologist and 51.1% by a medical officer. These findings highlights the need for well organized systematic screening programs for DR. Effective screening and treatment programs are known to reduce the burden of blindness due to diabetes, especially in vulnerable populations [27]. However, in limited resource settings such as in Sri Lanka with low access to specialist care, a suitable alternative could be to train primary care clinicians to recognize DR in at risk populations with a reasonable degree of accuracy [28].

In the Sri Lankan adult population with young-onset diabetes age, duration of diabetes, HbA1C and urine Microalbumin levels were significantly associated with the presence of DR, while HbA1c was also a significant predictor of severity. In addition, HbA1c had a stronger association with the occurrence of DR than FBG (Table 1). HbA1c has been described as a predictor of both micro- and macrovascular complications of diabetes and studies have shown a linear increase in the prevalence of DR when HbA1c is more than 6.5% (47.5 mmol/mol) [29]. This highlights the importance of regular HbA1c measurement and the need for targeted ophthalmological assessment in patients with higher HbA1c values. However, recent studies have shown that costlier laboratory investigations like HbA1c and urine Microalbumin are frequently missed benchmarks in diabetes care in Sri Lanka, as facilities are currently unavailable in the Government Health Sector [30]. Considering the finding that age, duration of diabetes and HbA1c all were significantly associated with DR, it can be concluded that long term poor glycaemic control is responsible for the observed increase in risk. However, it is important to appreciate that poor glycaemic control is a risk factor that is amenable to intervention. We also observed that patients with DR had a significantly higher degree of microalbuminuria and the degree of microalbuminuria was higher in those moderate DR than in those with mild DR. Studies have shown that irrespective of the age of onset, individuals with microalbuminuria are more likely to have retinopathy than those without microalbuminuria, even after controlling for potential confounders such as glycaemic control, hypertension and duration of diabetes [31]. In addition patients with microalbuminuria and DR have shown a more rapid decline in renal function in prospective follow up studies [32]. Therefore targeted screening for DR is recommended for all patients with microalbuminuria, so that treatment can be instituted in the required patients to prevent ocular morbidity/blindness [33].

We also observed a negative relationship between anthropometric measurements (weight, BMI, WC and hip circumference) and the presence of DR. This relationship has similarly been examined in numerous epidemiologic studies, but results have been inconsistent. The Diabetes Control and Complications Trial (DCCT) reported that high BMI was associated with DR [34]. The EURODIAB Study reported that the WHR was an independent risk factor for DR after ≥ 7 years of follow-up [35]. In contrast, Chaturvedi and Fuller, Dowse et al. and Lim, et al. reported that decreasing BMI is associated with a higher prevalence of DR [5,36,37]. Our results are in conformity with these latter studies. Leanness is probably an indicator of the severity of diabetes and long standing exposure to hyperglycemia. Another possible explanation is that persons with DR could have adopted positive behavioral modifications after diagnosis that has led to healthy anthropometric parameters.

There are several limitations to our study, the cross-sectional design of our study can only demonstrates an association between DR and identified risk factors, and limits the inference of causality. Therefore, it is important to conduct prospective studies in newly diagnosed young-onset adult patients with diabetes without DR and look for causality during subsequent follow up. The lack of fundus photographs is also a limitation; it is possible that we may have missed patients with early DR, resulting in the underestimation of rate. Retinal photography is the gold standard for the diagnosis of DR. In addition the grading given by the ophthalmologist was not validated by measuring intra/inter-observer concordance. However, ophthalmoscopy by experienced ophthalmologists also has been shown to have acceptable sensitivity and is useful in resource limited settings such as in Sri Lanka [38]. We were also unable to evaluate data on treatment, including insulin therapy and its’ association with DR. We recruited the present sample from patients referred from three large tertiary care hospitals in Sri Lanka, located in the most urban parts of the country, which could have led to a significant selection bias. Furthermore it is important to appreciate that clinic based data as in the context of the current study, over estimates the actual community prevalence and disease burden. Hence there is a need for population based studies evaluating complications in adult patients with young-onset diabetes.

## Conclusions

In this large multi center study nearly one in five adults with young-onset diabetes was found to have retinopathy, with a majority having only mild degrees of retinopathy. Age, duration of diabetes, HbA1C and urine Microalbumin levels were significantly associated with the presence of retinopathy, while HbA1c was also a significant factor determining severity. Nearly 50% of the study population has never undergone retinal screening by an ophthalmologist, highlighting the need for well organized screening programs. In addition there is also an urgent need for population based studies evaluating the true disease burden related to retinopathy in Sri Lankan adult patients with young-onset diabetes.

## Competing interests

The authors declare that they have no competing interests.

## Authors’ contributions

PK, MJ, NPW, RS and DRM made substantial contribution to conception and study design. MJ, NPW and PK were involved in data collection. YCW, PR, WMUAW, PK, RS and DRM were involved in refining the study design, statistical analysis and drafting the manuscript. YCW, PR, WMUAW and PK critically revised the manuscript. All authors read and approved the final manuscript.

## Pre-publication history

The pre-publication history for this paper can be accessed here:

http://www.biomedcentral.com/1472-6823/14/20/prepub

## Supplementary Material

Additional file 1 Diabetic retinopathy disease severity scale. Click here for file
