# Supplementary material for: Retinopathy among young adults with Diabetes Mellitus from a tertiary care setting in Sri Lanka
Source: BMC Endocr Disord. 2014 Mar 4;14:20. doi: 10.1186/1472-6823-14-20 (PMC3943575; doi:10.1186/1472-6823-14-20)
Supplement: Additional file 1 — Diabetic retinopathy disease severity scale. [file 1472-6823-14-20-S1.doc]

| **Disease Severity Level** | **Findings on Dilated Ophthalmoscopy** |
| --- | --- |
| No retinopathy | No abnormalities |
| Mild non-proliferative  diabetic retinopathy | Micro-aneurysms only |
| Moderate non-proliferative  diabetic retinopathy | More than just micro-aneurysms but less than severe non-proliferative diabetic retinopathy |
| Severe non-proliferative  diabetic retinopathy | Any of the following:  More than 20 intra-retinal haemorrhages in each of 4 quadrants; Definite venous beading in 2 quadrants;  Prominent intra-retinal microvascular abnormalities in 1 quadrant  And no signs of proliferative retinopathy |
| Proliferative  diabetic retinopathy | One or more of the following:  Neo-vascularization, vitreous/pre-retinal haemorrhages |
| Diabetic macular oedema  (Maculopathy) | Some apparent retinal thickening or hard exudates in posterior pole |

**Diabetic Retinopathy Disease Severity Scale**
